# Draft Genome Sequence of *Enterococcus mundtii* SL 16, an Indigenous Gut Bacterium of the Polyphagous Pest *Spodoptera littoralis*

**DOI:** 10.3389/fmicb.2016.01676

**Published:** 2016-10-25

**Authors:** Bosheng Chen, Chao Sun, Xili Liang, Xingmeng Lu, Qikang Gao, Pol Alonso-Pernas, Beng-Soon Teh, Alexey L. Novoselov, Wilhelm Boland, Yongqi Shao

**Affiliations:** ^1^Laboratory of Invertebrate Pathology, College of Animal Sciences, Zhejiang UniversityHangzhou, China; ^2^Analysis Center of Agrobiology and Environmental Sciences, Zhejiang UniversityHangzhou, China; ^3^Department of Bioorganic Chemistry, Max Planck Institute for Chemical EcologyJena, Germany

**Keywords:** *Enterococcus mundtii*, genome sequencing, symbiosis, *Spodoptera littoralis*, intestinal tract

## Introduction

Insects are the most abundant and diverse animal class on Earth, and they are associated with an amazing variety of symbiotic microorganisms, which participate in many relationships with the hosts (Douglas, 2015). For example, the fungal symbiont (*Leucoagaricus gongylophorus*) of leaf-cutting ants produces diverse enzymes for the degradation of plant material (Kooij et al., 2016). Similarly, *Bacillus pumilus* isolated from the gut of wood boring *Mesomorphus* sp. (Coleoptera: Tenebrionidae) exhibits significant cellulolytic and xylose isomerase activities (Balsingh et al., 2016).

The Lepidoptera, including moths and butterflies, is one of the most widespread and widely recognizable insect orders in the world. Although butterflies and moths play an important role in the natural ecosystem as pollinators and as food in the food chain, their leaf-chewing larvae are often problematic in agriculture, as their main source of food is live plants (Mithöfer and Boland, 2012). The leafworm *Spodoptera littoralis* (Lepidoptera: Noctuidae) is a highly polyphagous lepidopteran pest found worldwide and also an important model system used in a variety of biological research. Recent extensive surveys of its microbiome reveal that *Enterococcus mundtii* is one of the predominant gut microorganisms of *S. littoralis* and present at high frequency (Tang et al., 2012; Chen et al., 2016; Teh et al., 2016). Particularly, a stable isotope labeling-based approach suggested that this phylotype was also highly metabolically active inside the host across life history of *S. littoralis*, indicating the significant role played by *E. mundtii* in host biology (Shao et al., 2014). Therefore, the symbiotic *E. mundtii* probably constitutes a key factor for the success of this generalist herbivore in adapting to different environments and food sources. The aim of this study was to produce a genome sequence of the strain SL 16, which would assist in understanding of the coevolution of the microbe and the insect host. The dataset has been submitted to NCBI Whole Genome Shotgun (WGS) projects and is reported here, providing an overview of the genome sequence and relevant features of gut symbiotic *E. mundtii*.

## Materials and methods

### Isolation of the bacterial strain

*E. mundtii* strain SL 16 was isolated from the mature 5th instar larva using standard microbiology methods. Briefly, the normal larvae were washed and sedated on ice for at least 1 h to anesthetize them. Then the whole gut sections were dissected from larvae using a fine Vannas scissor and forceps under a binocular microscope (Shao et al., 2013). The fresh gut tissues were put into phosphate buffered saline (PBS: 137 mM NaCl, 10 mM Na_2_HPO_4_, 2 mM KH_2_PO_4_, and 2.7 mM KCl) and homogenized by hand with a sterile pestle. Bacterial isolates were made by plating the homogenized gut tissues on the *Enterococcus* Selective Agar (45183, Fluka). After incubation for 24 h at 30°C, the growing bacterial colonies were sub-cultured twice on the same agar medium. 2, 3, 5-Triphenyltetrazolium chloride (TTC) in the medium is reduced to insoluble formazan inside the bacterial cells, which gives pink or red coloration to enterococcal colonies. These purified enterococcal colonies were tested for key phenotypic traits including carbohydrate fermentation capability, motility, and pigment production as previously described (Manero and Blanch, 1999). Furthermore, the taxonomy was validated by colony PCR and sequencing of the amplified 16S rRNA gene. The representative *E. mundtii* isolate, designated strain SL 16, was selected for this WGS project.

Fluorescence in situ Hybridization (FISH) was applied to localize the dominant enterococci as previously described (Shao et al., 2014). Shortly, FISH was performed on 5 μm thin cross sections of the cold polymerizing resin (Technovit 8100, Heraeus Kulzer GmbH, Wehrheim, Germany) embedded gut tissue. The specificity of probes was tested and hybridization condition was achieved as described (Tang et al., 2012). The sample was hybridized with 1.5 mM FITC-labeled *Enterococcus*-specific probe in hybridization buffer containing 900 mM NaCl, 20 mM Tris-HCl (pH 8.0), 20% formamide, 1% SDS. And images were taken with an Axio Imager Z1 microscope (Carl Zeiss, Jena, Germany). For scanning electron microscopy (SEM), cells were fixed in paraformaldehyde (1%), and glutaraldehyde (0.25%), dehydrated by ascending alcohol series and dried. After coating samples with gold, scanning electron micrographs were taken with a LEO 1525 instrument (Carl Zeiss, Jena, Germany).

### Genomic DNA isolation, library preparation and sequencing

The genomic DNA was extracted from the cultured bacterium according to Pospiech and Neumann (1995). DNA quality was examined by 1% agarose gel electrophoresis and quantified using a NanoDrop™ spectrophotometer. The DNA library was constructed using the TruSeq™ DNA Sample Preparation Kit (Illumina Inc., San Diego, CA), and 5 μg of pure genomic DNA was prepared for a standard Illumina shotgun library construction. Briefly, genomic DNA was first sheared to a size ranging between 400 and 500 bp using the Covaris M220 per the manufacturer's recommendations. The fragmented DNA sample was end-repaired, dA-tailed, and ligated to multiplex adapters according to the manufacturer's instructions. The ligated products were purified and further enriched using PCR. The quality of the final amplified libraries were checked by running an aliquot (1 μL) on a high-sensitivity Bioanalyzer 2100 DNA Chip (Agilent Technologies). Paired-end sequencing was performed by using an Illumina MiSeq platform (Illumina Inc., San Diego, CA) at Majorbio Bio-pharm Technology Co., Ltd (Shanghai, China) according to the manufacturer's instructions (Zhang et al., 2016).

### Preprocessing and genome assembly

The quality of sequence reads was evaluated using the FastQC tool as previously described (Balsingh et al., 2016). Reads with >10% Ns and/or 25–35 bases of low quality (≤ Q20) were filtered out, and adapter and duplication contamination were removed as well as read ends were trimmed off. The filtered reads were assembled with Short Oligonucleotide Analysis Package (SOAP) *de novo* version 2.04 using a range of *k*-mer sizes (Li et al., 2009). Then GapCloser version 1.12 was used to close any internal gaps in the optimal scaffolded assembly. Repeats were predicted by RepeatMasker and Tandem Repeats Finder (TRF) tools (Rédou et al., 2016). Barrnap version 0.4.2 and tRNAscan-SE version 1.3.1 were employed to predict rRNAs and tRNAs respectively. The genome was annotated using Glimmer version 3.02 (Xu et al., 2014). The Clusters of Orthologous Groups of proteins (COG) categories were assigned to the SL 16 genome annotation using blastp (BLAST 2.2.28+) against the COG genes collection (Von Mering et al., 2005). The translations of the identified coding sequences (CDSs) were also used to search against the Protein family (Pfam) database with *E*-value cut-off of 1-e5. The metabolic pathway analysis was constructed using the Kyoto Encyclopedia of Genes and Genomes (KEGG) (Kanehisa et al., 2014).

## Interpretation of data set

### Whole genome sequencing of *E. mundtii* SL 16

**Figure 1:**
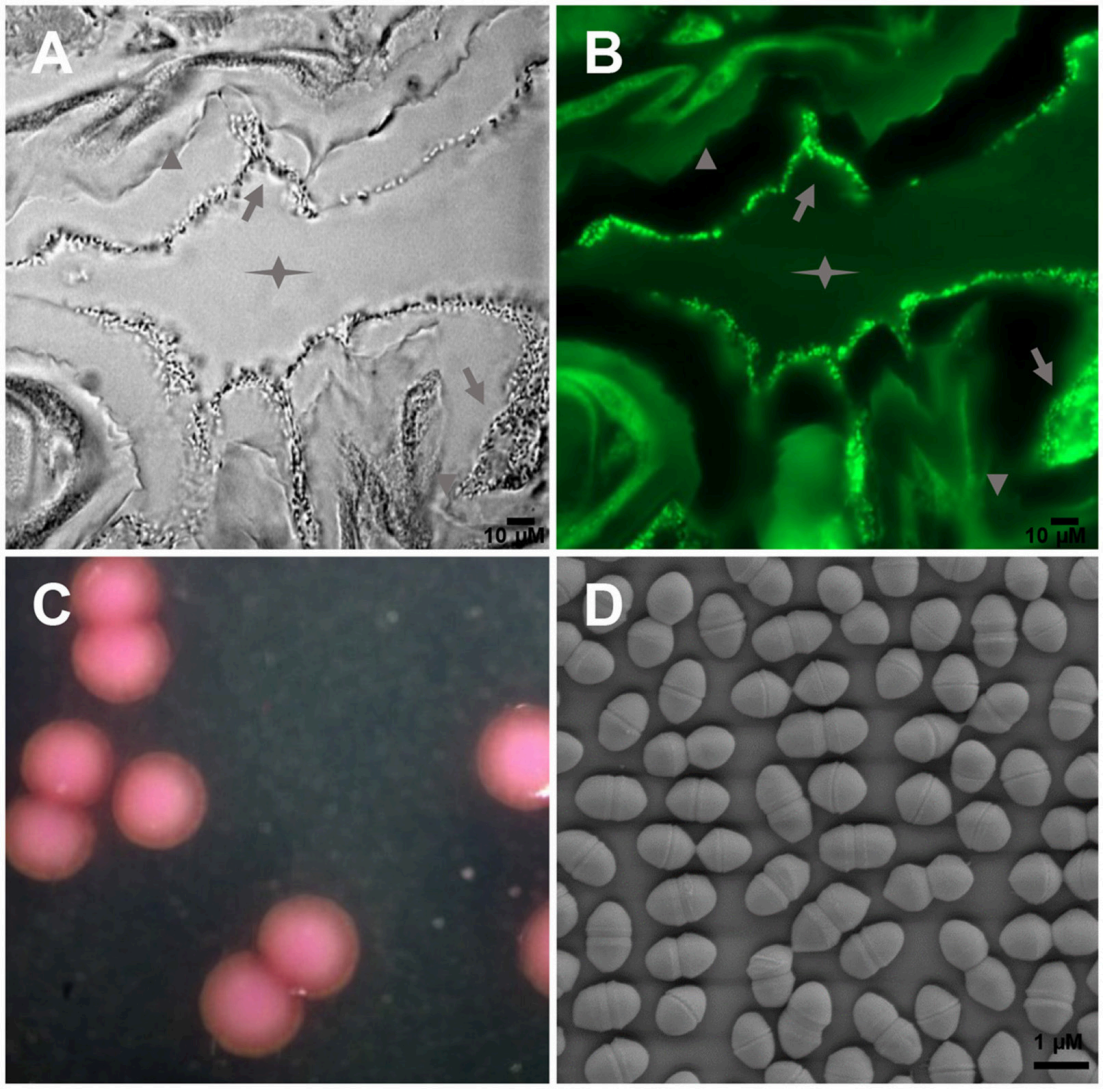
**Images of *E. mundtii* from *S. littoralis* reveal bacterial gut localization and phenotypic characteristics**. **(A)** Phase-contrast micrograph and **(B)** FISH with a FITC-labeled Enterococcus-specific probe (green) show a high density of bacterial cells adhere on the mucus layer lining the gut epithelium. Star indicates the gut lumen, arrowhead indicates the gut epithelium tissue, and arrow indicates bacteria. **(C)** Photomicrograph of source organism on Enterococcus selective agar. 2, 3, 5-Triphenyltetrazolium chloride (TTC) in the agar is reduced to insoluble formazan inside the bacterial cells, which gives pink or red coloration to colonies. **(D)** Scanning electron micrograph of *E. mundtii* SL 16, showing cell division.

Large amounts of *E. mundtii* closely adhere to the mucosal layer of *S. littoralis* gut epithelium, where they form a biofilm-like structure (Figures 1A,B). Strain SL 16 displays characteristic phenotypes of *E. mundtii*. It grows well on Slanetz and Bartley medium (Slanetz and Bartley, 1957), producing smooth, circular, glistening colonies (Figure 1C). The bacterial cells are 0.5–1.0 μm in diameter, and occur in the form of pairs (Figure 1D). Strain SL 16 could utilize various carbon sources, including xylose, cellobiose, and sucrose (Table 1).

**Table 1:** ***E. mundtii* SL 16 genome resources and characteristics**.

	**Name**	**Genome resources/characteristics**
1	NCBI Bioproject ID	PRJNA337899
2	NCBI Biosample ID	SAMN05513637
3	NCBI Genome Accession Number	MCRG00000000
4	Sequence type	Illumina Miseq
5	Total number of Reads	3,515,580
6	Overall coverage	>100x
7	Estimated genome size (bp)	3,296,585
8	GC content (%)	38.36
9	Average of gene length (bp)	889
10	Protein coding genes	2939
11	tRNA coding genes	59
12	Motility	Non-motile
13	Cellobiose metabolism	Positive
14	Xylose metabolism	Positive
15	Arabinose metabolism	Positive
16	Sucrose	Positive

Sequencing the genome of *E. mundtii* SL 16 produced a raw data set of 1,764,821,160 total bases. During the quality control, Illumina PCR adapter reads and low-quality reads were removed, and a total of 3,469,570 mate-pair reads (total bases 1,698,525,052 bp) were retained. The cleaned sequence reads were assembled with a *k*-mer setting of 125, which was determined by the optimal assembly result. The resulting genome sequence has an estimated size of 3,296,585 bp and a G+C content of 38.36%. 43,977 bp were repeats as predicted by RepeatMasker and TRF tools, which constituted 1.33% of the entire assembled genome.

A total of 3125 genes with sequence length of 2,780,928 bp were predicted, which account for 84.4% of the genome, and 59 tRNA genes were identified by tRNAscan-SE. CDSs were searched against the NR, GO, string, Swiss-Prot, COG, and KEGG databases to analyze gene functions and metabolic pathways. In all, 1493 CDSs were assigned to COG families and 1411 CDSs were included in 154 pathways. Several physiological traits that may explain the successful adaptation of this bacterium to the environment of the gut have been found. In particular, a large amount of the coding capacity encountered in the genome of SL 16 (almost 12%) is dedicated to genes assigned to functions related to carbohydrate transport and metabolism, which matches well with the observed physiological characteristics of this strain (Table 1). This feature is shared with other colonic inhabitants, such as *Bacteroides fragilis* (Flint et al., 2008), and reflects the ecological niche of the organism presented inside a herbivore gut. The genome encodes several ABC-type sugar transporters, sugar-binding proteins, and a rich suite of glycosyl hydrolases, such as β-N-acetylhexosaminidase, α-galactosidase, β-glucosidase, β-galactosidase, and α-glucosidase. Moreover, the pyruvate dissipation pathways predicted for SL 16 include the capacity to produce L-lactate and several other fermentation metabolites, like short-chain fatty acids formate and acetate. This metabolic flexibility is expected to aid in efficient digestion and conversion of plant saccharides, thus promoting host development.

In conclusion, here we report a 3.30 Mbp draft genome sequence of *E. mundtii* strain SL 16, isolated from the generalist herbivore *S. littoralis*. The final *de novo* assembly is based on 1765 Mbp of Illumina data which provides an average coverage of 535 ×. Analysis of the genome shows high correlation between the genotypes and the phenotypes.

### Direct link to deposited data and information to users

The dataset submitted to NCBI include the assembled consensus sequence of *E. mundtii* SL 16 in Fasta format. The genome sequence can be accessed at DDBJ/EMBL/GenBank under the accession no. MCRG00000000. This paper describes the first version of the genome (https://www.ncbi.nlm.nih.gov/nuccore/MCRG00000000).

## Author contributions

Work was planned by YS and WB, and executed jointly by BC and CS. XLi and BT were associated with isolation of the bacterium. AN and PA performed bioinformatics analyses. QG and XLu contributed to the DNA sequencing.

### Conflict of interest statement

The authors declare that the research was conducted in the absence of any commercial or financial relationships that could be construed as a potential conflict of interest.
